# Synovial *SLC1A5*-Associated Amino Acid Metabolic Features in Knee Osteoarthritis: An Integrated Analysis of Synovial Transcriptomics, Fecal Metabolomics, and Gut Microbiota

**DOI:** 10.3390/metabo16100764

**Published:** 2026-10-12

**Authors:** Xingzong Zhu, Hongrui Du, Bin Li, Hangyuan He, Yang Tan

**Affiliations:** Department of Joint Surgery and Sports Medicine, Zhongnan Hospital of Wuhan University, Wuhan 430071, China

**Keywords:** knee osteoarthritis, synovium, *SLC1A5*, amino acid metabolism, nitrogenous peptides, *Bacteroides*, gut microbiota, multi-omics

## Abstract

**Background:** This exploratory study aimed to characterize amino acid related features in osteoarthritis (OA). **Methods:** We analyzed independent public synovial transcriptomic datasets and paired fecal metabolomics and 16S ribosomal RNA profiles from 20 patients with knee OA and 14 controls. Metabolite-sensing genes were intersected with nominally differential genes and prioritized using elastic net, support vector machine recursive feature elimination, and extreme gradient boosting. **Results:** *SLC1A5* (solute carrier family 1 member 5) expression was lower in the discovery cohort GSE89408 (log2 fold change = −0.532, *p* = 0.0159), but did not meet the false discovery rate (FDR) threshold of 0.05 (FDR = 0.0697). GSE55235 showed a concordant decrease (log2 fold change = −0.695, *p* = 0.00536; area under the receiver operating characteristic curve = 0.830). Single-cell computational predictions from three OA donors suggested endothelial, smooth muscle, and mast-cell contexts. Tissue-level *SLC1A5* expression correlated with inferred M2 macrophage proportions after group adjustment (r = −0.575, FDR = 0.002). Six features with Level 3 putative glutamine-, asparagine-, or glutamate-containing tripeptide annotations differed between groups (q = 0.00245 to 0.00948), whereas the corresponding free amino acids did not reach FDR significance. *Bacteroides* showed a lower centered log-ratio value in OA (q = 0.00273); none of its six tripeptide correlations remained significant after group adjustment. **Conclusions:** These observations generate a hypothesis about intestinal amino acid-related features and synovial *SLC1A5* expression; they do not establish a direct gut-joint relationship, peptide identity, or a metabolic mechanism.

## 1. Introduction

Osteoarthritis (OA) is a heterogeneous disease involving the synovium, cartilage, subchondral bone, and other joint tissues. Mechanical loading, ageing, low-grade inflammation, and systemic metabolic status all contribute to its development and progression [1,2]. Associations of obesity, insulin resistance, and metabolic syndrome with OA have advanced the concept of metabolic OA, while most mechanistic work has centered on altered glycolysis, lipotoxicity, and dysregulated lipid mediators. Amino acid metabolism is another fundamental determinant of cellular homeostasis. Beyond protein synthesis, amino acids support anaplerosis, nucleotide synthesis, glutathione production, redox balance, mechanistic target of rapamycin (mTOR) signaling, and immune activation. The chronically inflamed, hypoxic, and oxidatively stressed OA synovium may therefore be sensitive to changes in amino acid uptake, exchange, and utilization. This perspective links systemic nutritional status, intestinal metabolism, and local synovial pathology within one metabolic framework.

The synovium lies at the intersection of local metabolic and inflammatory signaling in OA. Synovial fibroblasts, macrophages, endothelial cells, and other resident cells reshape the joint microenvironment through nutrient competition, metabolic adaptation, and intercellular communication [3]. *SLC1A5* (solute carrier family 1 member 5) encodes the neutral amino acid transporter alanine, serine, cysteine transporter 2 (ASCT2) and mediates the transport of substrates including glutamine, asparagine, and serine. Its activity is closely linked to nutrient acquisition, mTOR complex 1 (mTORC1) activation, energy metabolism, and redox homeostasis [4,5]. *SLC1A5* is therefore a biologically plausible candidate for examining how synovial amino acid uptake relates to the inflammatory microenvironment.

The gut microbiota contributes to dietary and endogenous protein processing and amino acid metabolism. Clinical studies have reported altered gut microbial composition in OA [6,7,8,9]. Fecal free amino acids reflect several competing influences, including dietary input, microbial and host metabolism, absorption, and intestinal transit. Features putatively annotated as short peptides may provide complementary descriptive information, but cannot by themselves quantify proteolysis or amino acid flux. We therefore examined free glutamine (Gln), asparagine (Asn), and glutamate (Glu) alongside features assigned Gln/Asn/Glu-containing tripeptide names. Some *Bacteroides* species have experimentally characterized proteolytic or peptide-related capabilities [10,11,12]; genus-level abundance does not establish these activities in a clinical sample. Previous fecal metabolomics work in obesity-related OA provides a rationale for examining peptide-associated features [6]. These observations motivated an exploratory comparison of synovial amino acid transport-related expression and clinical fecal profiles.

In the present study, we analyzed two complementary but independent components. Public synovial bulk transcriptomes, single-cell computational communication predictions, three machine-learning algorithms, and a second bulk cohort were used to prioritize candidate genes and describe their cellular and immune context. In a separate clinical cohort, paired fecal metabolomics and 16S ribosomal RNA (rRNA) profiles were analyzed to describe amino acid-related features and their associations with *Bacteroides*. The cross-source comparison was intended to generate hypotheses, without assuming that the fecal and synovial findings were linked within individual participants.

## 2. Materials and Methods

### 2.1. Study Design

**Figure 1:**
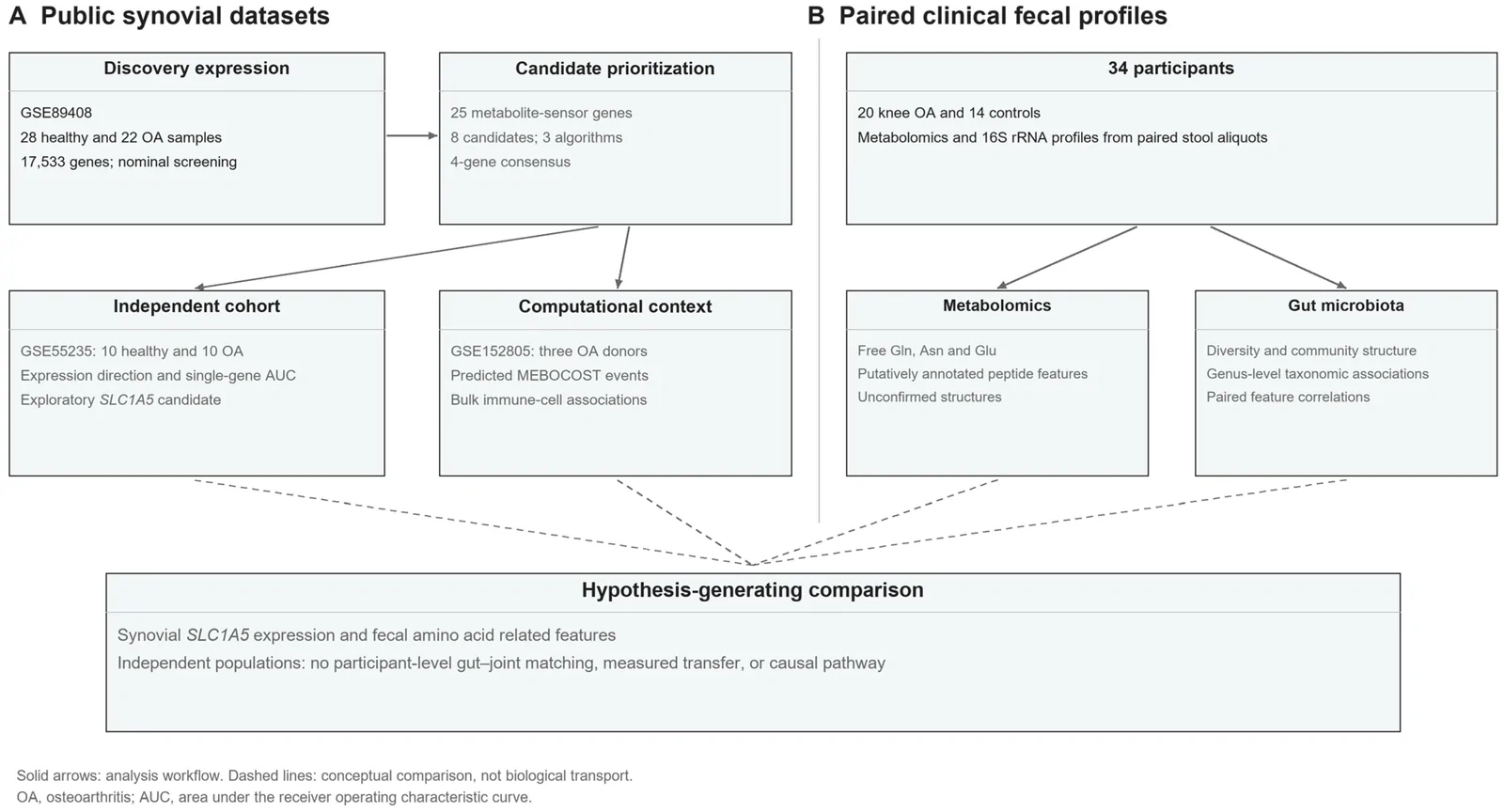
**Study design and analysis workflow.** (**A**) Public synovial datasets were used for bulk differential expression, metabolite-sensing candidate selection, three-model consensus analysis, independent-cohort expression assessment, and single-cell contextualization. (**B**) Untargeted metabolomics and 16S rRNA sequencing were performed on the same fecal samples from 34 participants in the clinical cohort. The two evidence streams were compared conceptually around synovial *SLC1A5* and fecal amino acid-related features, without participant-level gut–joint matching.

This study used two independent data sources (Figure 1). Public synovial datasets GSE89408, GSE55235, and GSE152805 were used for candidate prioritization and cellular contextualization; the analysis stages are summarized in Table 1. The clinical case–control cohort included 20 participants with knee OA and 14 controls, whose baseline characteristics are summarized in Table 2 and Table S1. Metabolomics and 16S rRNA sequencing were performed on paired fecal aliquots. Participant-level integration was restricted to the two fecal platforms. Cross-source biological interpretation meant comparing the known biological role of a synovial candidate with fecal feature classes and within-cohort associations. It did not involve sample pooling, a cross-tissue correlation test, mediation analysis, or inference of metabolite transfer. Reporting was guided by Strengthening the Reporting of Observational Studies in Epidemiology (STROBE), Strengthening The Organization and Reporting of Microbiome Studies (STORMS), and the Metabolomics Standards Initiative (MSI) [13,14,15].

### 2.2. Public-Data Analysis: Synovial Bulk Transcriptomics and Candidate-Gene Selection

GSE89408 and GSE55235 were obtained from the Gene Expression Omnibus (GEO). GSE89408 comprised 28 healthy and 22 OA synovial samples. Differential expression across 17,533 genes was performed using filterByExpr and trimmed mean of M-values (TMM) normalization in edgeR (empirical analysis of digital gene expression data in R) 4.0.16, followed by voom, lmFit, and eBayes in limma (linear models for microarray data) 3.58.1. Twenty-five metabolite-sensing genes identified by MEBOCOST, a tool for metabolic cell–cell communication modeling using single-cell transcriptomes, were intersected with 5189 nominally differential genes defined by *p* < 0.05 and |logFC| > 0.5, yielding eight candidates. These candidates entered elastic net, support vector machine recursive feature elimination (SVM-RFE), and extreme gradient boosting (XGBoost). Elastic net used five-fold cross-validation and retained the five genes with the largest absolute coefficients; SVM-RFE used ten-fold cross-validation and retained the five highest-ranked genes; XGBoost used five-fold cross-validation and retained features with Gain > 0. Genes shared by all three models formed the consensus set. GSE55235, comprising 10 healthy and 10 OA synovial samples, was used to evaluate expression direction and discriminatory performance [16,17]. Probe 208916_at represented *SLC1A5*, and the 95% confidence interval (CI) for the area under the receiver operating characteristic (ROC) curve (AUC) was estimated using 20,000 stratified bootstrap resamples. Elastic net is penalized regression combining L1 and L2 penalties; SVM-RFE ranks features by recursive elimination, and XGBoost is a gradient-boosted tree method. filterByExpr filters low-expression genes; voom estimates precision weights, lmFit fits linear models, and eBayes moderates variance estimates. Here logFC denotes log2 fold change. The discovery screen used nominal *p* values and was exploratory. Its feature preselection preceded model cross-validation; therefore, cross-model agreement does not constitute independent validation, and the reported cross-validation does not estimate the performance of a fully nested selection pipeline. The external AUC describes a single gene in 20 samples, rather than an externally validated multivariable classifier. As a transparent additional calculation, Benjamini–Hochberg correction across the four consensus-gene *p* values in GSE55235 gave q = 0.01072 for *SLC1A5*; this is a four-candidate adjustment, not transcriptome-wide FDR.

### 2.3. Public-Data Analysis: OA Synovial Single-Cell Data, Metabolite-Mediated Communication, and Immune Associations

The original GSE152805 study included paired synovial and cartilage samples. We analyzed three OA synovial samples (GSM4626763, GSM4626764, and GSM4626765). Seurat (single-cell analysis software) objects were generated in R 4.3.3 using Seurat 4.4.0 and SeuratObject 4.1.4. Cells with 200–6000 detected features and mitochondrial transcript proportions ≤ 20% were retained, resulting in 10,547 cells. Data were normalized with LogNormalize, 2000 highly variable genes were selected, and the first 20 principal components were used for neighbor-graph construction, uniform manifold approximation and projection (UMAP), and clustering at a resolution of 0.3. Cell identities followed the nomenclature of the original study together with canonical markers and cluster markers [18]. MEBOCOST 1.2 was run with cutoff_prop = 0.15, n_shuffle = 1000, min_cell_number = 10, permutation_test_fdr, and pval_cutoff = 0.05 [19]. We examined the receivers, metabolites, and sensor_in_receiver expression intensity of *SLC1A5*-related events. Immune-cell proportions in GSE89408 were inferred with CIBERSORT (cell-type identification by estimating relative subsets of RNA transcripts), a bulk-expression deconvolution method. Spearman correlations were calculated between *SLC1A5* and immune-cell proportions; the ranks of both variables were then residualized against OA/healthy group to obtain group-adjusted partial correlations, with 5000 permutations and Benjamini–Hochberg false discovery rate (FDR) correction [20]. SeuratObject stores single-cell data, and LogNormalize performs library-size normalization followed by log transformation. MEBOCOST predicts metabolite–sensor events from expression-based information; sensor_in_receiver is the model-reported sensor expression in the receiver population, not measured transport or metabolite uptake. The biological sample size was three OA donors, not 10,547 independent replicates. The pooled analysis lacked healthy single-cell controls and donor-level replication or leave-one-donor-out validation; its cell-context results are descriptive.

### 2.4. Clinical Cohort: Participants and Fecal Samples

The local case–control cohort comprised 34 participants, including 20 patients with knee osteoarthritis (OA) and 14 non-OA controls. Knee OA was diagnosed according to the American College of Rheumatology (ACR) clinical and/or radiographic criteria, and radiographic severity was evaluated using the Kellgren–Lawrence (K-L) grading system. Patients with a K-L grade ≥ 2 were considered to have radiographic knee OA. Control participants were recruited from individuals undergoing routine health examinations/patients without clinical evidence of knee OA at Zhongnan Hospital of Wuhan University, and the absence of knee OA was assessed by clinical evaluation and knee radiography.

Participants were eligible if they were adults, were able to provide a fresh fecal sample, and had complete clinical information available for analysis. Participants were excluded if they had inflammatory or autoimmune joint diseases, gastrointestinal diseases, active infection, malignant tumors, severe hepatic or renal dysfunction, or a history of major gastrointestinal surgery. To minimize potential confounding of the gut microbiota, individuals who had received systemic antibiotics, probiotics, prebiotics, or other microbiota-modulating preparations within the preceding 4 weeks were excluded. Information regarding recent medication use, including glucose- and lipid-lowering medications, as well as dietary habits and relevant comorbidities, was collected using medical records and/or a standardized questionnaire. Demographic and clinical characteristics, including age, sex, body mass index (BMI), comorbidities, K-L grade, and other clinical phenotypes, are summarized in Table 2. The study protocol was approved by the Medical Ethics Committee of Zhongnan Hospital of Wuhan University (approval no. 20240819), and all participants provided written informed consent before enrollment.

Fresh fecal samples were collected in the morning before breakfast or before initiation of study-related treatment, using sterile fecal collection containers, while avoiding contamination with urine or toilet water. Samples were transferred to the laboratory within 2 h of collection under refrigerated conditions (approximately 4 °C) and immediately processed. Each sample was homogenized and divided into paired aliquots for 16S rRNA gene amplicon sequencing and untargeted metabolomic profiling, respectively. Aliquots were immediately stored at −80 °C until analysis. Identical participant-specific sample identifiers were used for the microbiome and metabolomics datasets to ensure one-to-one matching across the two platforms.

### 2.5. Clinical Cohort: 16S rRNA Amplicon Sequencing

Microbial deoxyribonucleic acid (DNA) was extracted from fecal samples using the cetyltrimethylammonium bromide/sodium dodecyl sulfate (CTAB/SDS) method. Wilson’s bacterial genomic DNA extraction protocol provides a methodological reference for the CTAB-based approach [21]. In the cited protocol, protease digestion is followed by CTAB-assisted removal of polysaccharides and residual proteins [21]. The V4 region of the 16S rRNA gene was amplified with primers 515F/806R and sequenced as paired-end 250-base-pair (bp) reads on an Illumina NovaSeq 6000 (Illumina, San Diego, CA, USA). The 515F/806R primers were synthesized by Wuhan Tianyi Huayu Gene Technology Co., Ltd. (Wuhan, China) using an oligonucleotide synthesizer, and their sequence consistency with the intended designs was verified by the supplier. Quality control, read merging, and chimera identification were performed with fastp 0.23.4, FLASH 1.2.11, and VSEARCH 2.27.1. Amplicon sequence variants (ASVs) were generated using Quantitative Insights Into Microbial Ecology 2 (QIIME 2) 2024.5 and Deblur, and taxonomic annotation was performed in Mothur 1.48 against SILVA 138.1 [22,23]. Alpha diversity was described using observed ASVs and the Shannon index. Beta diversity was evaluated using Bray–Curtis distances, visualized by principal coordinate analysis (PCoA), and tested by permutational multivariate analysis of variance (PERMANOVA). Genus-level relative-abundance differences were adjusted using the Benjamini–Hochberg method. The main microbiome analyses were conducted in R 4.3.2. fastp performs read-quality filtering, FLASH (Fast Length Adjustment of SHort reads) merges read pairs, VSEARCH performs sequence searches and chimera checks, and Deblur denoises amplicon sequences. Mothur is a microbial community analysis package; SILVA is a curated ribosomal RNA reference database. Study-specific extraction parameters and the exact 515F/806R primer sequences were unavailable for this revision, limiting complete methodological reproducibility.

### 2.6. Clinical Cohort: Untargeted Fecal Metabolomics

For each fecal sample, 20 mg (±1 mg) was mixed with 400 μL of 70% methanol containing internal standards, vortexed for 3 min, sonicated in ice water for 10 min, vortexed for another 1 min, and incubated at −20 °C for 30 min. After centrifugation at 12,000 r/min for 10 min at 4 °C, the supernatant was transferred and centrifuged again for 3 min; 200 μL was used for analysis. Separation was performed on a Vanquish ultrahigh-performance liquid chromatography system coupled to a Q Exactive HF-X high-resolution mass spectrometer using a Waters ACQUITY Premier HSS T3 column (1.8 μm, 2.1 × 100 mm). Mobile phase A was water with 0.1% formic acid and mobile phase B was acetonitrile with 0.1% formic acid. The flow rate was 0.4 mL/min, the column temperature was 40 °C, and the injection volume was 3 μL. The 10-min gradient used 5% B at 0 min, 20% at 2 min, 60% at 5 min, 99% at 6–7.5 min, and 5% at 7.6–10 min. In positive and negative ion modes, the full-scan mass spectrometry (MS1) scan range was mass-to-charge ratio (*m*/*z*) 70–1000 at a resolution of 60,000; tandem mass spectrometry (MS2) resolution was 15,000 with collision energies of 30, 40, and 50 V. Raw data were converted to mzML with ProteoWizard, and XCMS was used for peak extraction, alignment, and retention-time correction. Missing peaks were imputed using k-nearest neighbors (KNN) and a one-fifth minimum-value rule, and support vector regression was used to correct signal drift. Features with an integrated annotation score >0.5 and a quality-control (QC) coefficient of variation <0.30 were retained. After positive- and negative-mode merging, 5967 annotated features were available, of which 1768 quality-controlled metabolites entered the report-based group comparison. ProteoWizard converts mass-spectrometry data to the open mzML format, and XCMS is a chromatographic peak-processing software package. Peak areas are relative analytical signals rather than absolute fecal concentrations.

Overall metabolic structure was visualized with principal component analysis (PCA) and orthogonal partial least squares discriminant analysis (OPLS-DA). Model performance was described by R^2^X(cum), R^2^Y(cum), and Q^2^(cum) and evaluated using 200 label permutations. The main analysis used Student’s *t*-test results provided for the 1768 quality-controlled metabolites and reported log2FC, *p* values, and Benjamini–Hochberg q values. Two exploratory layers of analysis were considered around *SLC1A5*-related amino acid metabolism. Free glutamine, asparagine, and glutamate described the fecal free-amino-acid reference pool, whereas features with putative tripeptide annotations provided a complementary descriptive profile. Tripeptides were selected by a structural rule requiring a name composed of three standard three-letter amino acid codes and at least one Gln, Asn, or Glu residue. The main analysis included 24 features assigned Level 3 putative tripeptide annotations, and Level 4 candidates were retained for exploratory reporting. Gln and Asn represent neutral amino acid substrates related to *SLC1A5*, while Glu connects glutamine catabolism, nitrogen transfer, and redox metabolism. The annotation levels and candidate names are retained from the analytical report. Level 3 does not confirm an exact peptide sequence, and Level 4 candidates remain unidentified features; these labels do not establish a specific structure under MSI criteria [15]. No confirmation with authentic standards, matched retention times, or targeted liquid chromatography–tandem mass spectrometry (LC–MS/MS) was performed. R^2^X and R^2^Y describe explained predictor and response variance, and Q^2^ describes cross-validated predictive ability; cum indicates cumulative values. A high R^2^Y alone does not demonstrate generalization. The cross-validation configuration and complete preprocessing within resampling are not documented in the available model report, and no independent clinical validation set was available.

### 2.7. Clinical Cohort: Paired Microbiota–Metabolite Integration

*Bacteroides* relative abundance and peak areas for the six FDR-significant Gln/Asn/Glu-related tripeptides were matched one-to-one across all 34 participants. Spearman rank correlations first described their joint variation across the complete case–control sample. *Bacteroides* ranks and tripeptide peak-area ranks were then separately residualized against OA/control group, and partial rank correlations were calculated to assess within-group continuous associations. Benjamini–Hochberg correction was applied separately to the six unadjusted and six group-adjusted tests. The unadjusted analysis captured common separation of OA-associated microbial and metabolite phenotypes, whereas the adjusted analysis quantified the residual within-group association. These exploratory tests were restricted to metabolites selected for case–control differences in the same cohort; the unadjusted correlations are therefore vulnerable to selection effects and shared group separation.

### 2.8. Statistical Analysis

Continuous variables were summarized as means with standard deviations (SD) or medians with interquartile ranges (IQR), according to their distributions, and categorical variables as counts and percentages. The metabolomics main analysis used Student’s *t*-test and reported log2 fold change (log2FC). Alpha diversity was compared with the Wilcoxon rank-sum test, and Bray–Curtis distance matrices were tested by PERMANOVA. *Bacteroides*–tripeptide associations were assessed using Spearman correlations and group-adjusted partial rank correlations. Benjamini–Hochberg correction controlled the false discovery rate across high-dimensional metabolite and genus analyses and across the six directed correlation tests separately for unadjusted and adjusted analyses. Two-sided *p* < 0.05 indicated nominal significance, and q < 0.05 indicated significance after multiple-testing correction. Report-based analyses were performed in R 4.3.2, and robustness analyses were conducted in Python 3.12.13. Main metabolite q values refer to 1768 tests; full-feature sensitivity q values refer to 5967 tests. The supplied genus report contains 290 taxonomic entries, whereas the centered log-ratio sensitivity analysis is restricted to 113 named genera. R is the statistical computing environment, and Python is the programming language used for sensitivity calculations.

### 2.9. Robustness and Sensitivity Analyses

The metabolomics sensitivity analysis began with all 5967 annotated features. Zero values were replaced by half the minimum positive peak area for each feature, followed by log2 transformation, Welch’s *t*-test, and Benjamini–Hochberg correction across all 5967 tests. The compositional genus-level sensitivity analysis included 113 named genera. Half the smallest nonzero relative abundance in the complete table was used as a pseudocount before centered log-ratio (CLR) transformation, followed by Welch’s *t*-test and FDR correction across 113 genera. Bray–Curtis PERMANOVA and permutational analysis of multivariate dispersions (PERMDISP) used 9999 permutations (seed = 46,106). These analyses evaluated the stability of the six core tripeptides, *Bacteroides*, and community-structure findings under alternative transformations and statistical assumptions.

## 3. Results

### 3.1. Public-Data Results: Multi-Step Screening Prioritized SLC1A5

**Figure 2:**
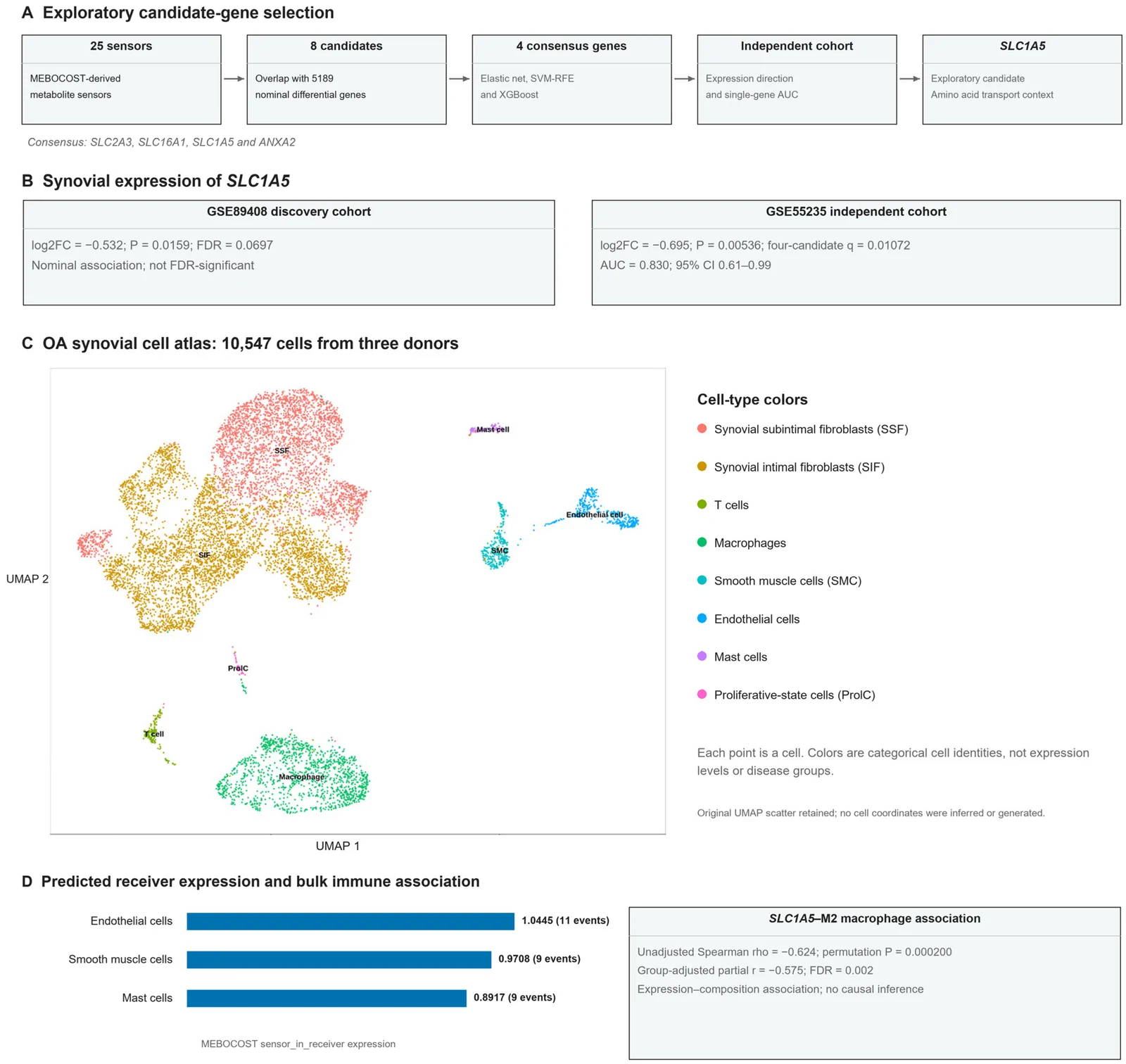
**Exploratory candidate screening and computational cellular context of *SLC1A5*.** (**A**) Candidate selection and independent-cohort expression assessment. (**B**) Synovial expression; the discovery association does not pass FDR < 0.05. The GSE55235 *p* value is 0.00536; correction across the four consensus genes gives q = 0.01072. (**C**) UMAP representation of three OA donors. Each point is a cell; colors distinguish annotated cell populations, not expression magnitude or disease groups. Salmon indicates synovial subintimal fibroblasts (SSF), ochre synovial intimal fibroblasts (SIF), olive T cells, green macrophages, turquoise smooth muscle cells (SMC), blue endothelial cells, purple mast cells, and pink proliferative-state cells (ProlC). These colors have no quantitative scale. (**D**) Model-reported sensor_in_receiver expression and the tissue-level correlation with inferred M2 macrophage proportions (Table S11); neither panel demonstrates transport activity or cell–cell causality. Panel C retains the supplied UMAP scatter and uses a larger layout and an expanded cell-type key; no new cell coordinates were generated.

Intersecting 25 MEBOCOST metabolite-sensing genes with 5189 nominally differential genes in GSE89408 yielded eight candidates: *ANXA2*, *LRRC8C*, *PMVK*, *RARG*, *SLC16A1*, *SLC1A5*, *SLC2A3*, and *SLC44A1*. Elastic net retained *ANXA2*, *SLC16A1*, *SLC1A5*, *SLC2A3*, and *SLC44A1*; SVM-RFE retained *ANXA2*, *PMVK*, *SLC16A1*, *SLC1A5*, and *SLC2A3*; and XGBoost retained seven candidates, excluding *PMVK*. *SLC2A3*, *SLC16A1*, *SLC1A5*, and *ANXA2* were shared by all three models (Figure 2A; Table S8). In GSE55235, *SLC1A5* and *ANXA2* changed in the same direction as in GSE89408. *SLC1A5* was prioritized for subsequent analyses because it encodes a neutral amino acid transporter and its predicted MEBOCOST events provided an amino acid-related context.

*SLC1A5* expression was lower in OA synovium in GSE89408 (logFC = −0.532, *p* = 0.0159, FDR = 0.0697; AUC = 0.680); the discovery result was nominally significant but did not pass FDR < 0.05. GSE55235 showed a concordant change (logFC = −0.695, *p* = 0.00536; AUC = 0.830, 95% CI 0.61–0.99) (Table 3; Figure 2B; Tables S8 and S9). Correction across the four consensus candidates gave *SLC1A5* q = 0.01072. The wide AUC interval and exploratory discovery selection limit claims about predictive performance.

### 3.2. Public-Data Results: Synovial Cellular Context and Immune Associations of SLC1A5

After quality control, 10,547 cells from three OA synovial samples in GSE152805 were retained and annotated as 4710 synovial intimal fibroblasts (SIFs) (44.7%), 3398 synovial subintimal fibroblasts (SSFs) (32.2%), 1371 macrophages (13.0%), 382 endothelial cells (3.6%), 343 smooth muscle cells (3.3%), 174 T cells (1.6%), 120 mast cells (1.1%), and 49 proliferative-state cells (0.5%) (Figure 2C). SIFs and SSFs were the dominant populations, followed by macrophages and smaller vascular and immune compartments.

MEBOCOST predicted 404 events passing its significance threshold, including 38 *SLC1A5*-related events involving L-glutamine, L-serine, L-asparagine, and L-glutamic acid. Endothelial-cell receivers accounted for 11 events and had the highest sensor_in_receiver intensity (1.0445). Smooth muscle cells and mast cells each accounted for nine events, with intensities of 0.9708 and 0.8917, respectively (Figure 2D; Table S10). These are expression-based computational receiver contexts and do not demonstrate amino acid sensing activity or flux. A predicted L-glutamic acid event does not establish direct glutamate transport by ASCT2.

At the tissue level, *SLC1A5* expression correlated inversely with the inferred proportion of M2 macrophages (Spearman ρ = −0.624; permutation *p* = 0.000200). The association remained significant after adjustment for OA/healthy group (r = −0.575, permutation FDR = 0.002) (Figure 2D; Table S11). This is an expression–composition association based on computational deconvolution, not evidence that *SLC1A5* regulates macrophage polarization.

### 3.3. Clinical-Cohort Results: Fecal Features with Putative Gln/Asn/Glu Tripeptide Annotations

After quality control, signal correction, and annotation filtering, 1768 metabolites entered the main OA-versus-control analysis (Table S2), and 347 reached FDR < 0.05 (Table S3). The reported OPLS-DA model had R^2^X(cum) = 0.422, R^2^Y(cum) = 0.998, and Q^2^(cum) = 0.648. The original report recorded zero Q^2^ exceedances and one R^2^Y exceedance among 200 label permutations. Using the finite-permutation convention (b + 1)/(B + 1), these counts correspond to *p* = 0.00498 and *p* = 0.00995, respectively. These within-cohort permutation summaries do not exclude overfitting, particularly given the near-perfect R^2^Y and small cohort; no independent predictive validation is claimed. Structural-name screening identified 24 Level 3 candidate tripeptide features (Table S4), of which nine were higher and 15 lower in the main analysis; Level 4 candidates are reported separately in Table S5.

**Figure 3:**
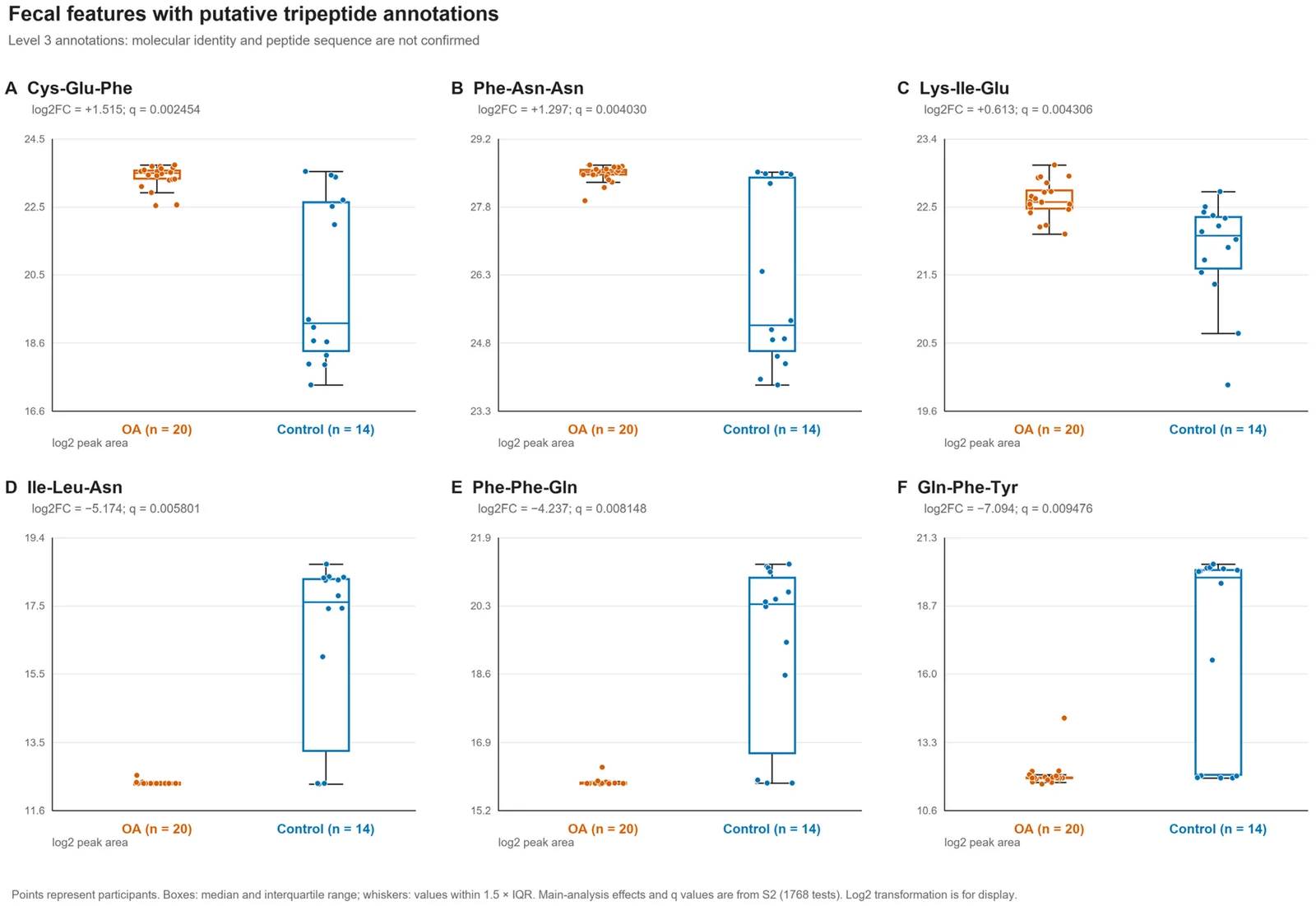
**Participant-level distributions of six features with Level 3 putative Gln/Asn/Glu tripeptide annotations.** Plots were drawn from the 34 sample columns in Table S2. Zero values were replaced by half the minimum positive value for each feature before log2 transformation for display. Each point is one participant; horizontal jitter is for visibility only. Boxes show the median and interquartile range, and whiskers extend to observations within 1.5 times the interquartile range. Effect estimates and q values derive from the main analysis of 1768 metabolites, rather than tests of the displayed log2 values. Orange indicates OA (n = 20) and blue indicates controls (n = 14). Candidate names do not establish confirmed peptide identity or sequence.

Six of the 24 target features reached FDR < 0.05. Cys-Glu-Phe (log2FC = 1.515, q = 0.002454), Phe-Asn-Asn (log2FC = 1.297, q = 0.004030), and Lys-Ile-Glu (log2FC = 0.613, q = 0.004306) were higher in OA, whereas Ile-Leu-Asn (log2FC = −5.174, q = 0.005801), Phe-Phe-Gln (log2FC = −4.237, q = 0.008148), and Gln-Phe-Tyr (log2FC = −7.094, q = 0.009476) were lower (Table 4; Figure 3; Table S2). The full 5967-feature log2-Welch sensitivity analysis preserved the direction of all six effects, with log2FC values of 3.046, 2.359, 0.741, −3.924, −3.220, and −4.875 and q values of 0.00644, 0.00697, 0.02143, 0.00367, 0.00373, and 0.00711, respectively. These sensitivity results are listed in Tables S4 and S12. The three-higher/three-lower pattern describes changes in putatively annotated signals; it cannot identify peptide sequences or distinguish proteolysis, transport, and microbial utilization.

In the main analysis, free glutamine, glutamate, and asparagine had OA-versus-control log2FC values of 0.219, 0.146, and 0.015 and q values of 0.293, 0.481, and 0.960, respectively. These values are reported in the “Log2FC” and “FDR” columns of Table S2 under Glutamine, Glutamic acid, and Asparagine, respectively; main-analysis q values were obtained by Benjamini–Hochberg correction across 1768 metabolites. In the log2-transformed Welch sensitivity analysis, their corresponding q values were 0.342, 0.587, and 0.827, as reported in the “Welch_FDR” column of Table S12 after correction across 5967 features. These results concern the three free amino acids, rather than the six putatively annotated tripeptide features. None of the three free amino acids met FDR < 0.05 in either analysis. The contrast with the six annotated peptide features is descriptive and does not establish altered peptide-bound amino acid availability, transport, or nitrogen flux.

### 3.4. Clinical-Cohort Results: Gut Microbial Diversity, Community Structure, and Bacteroides

**Figure 4:**
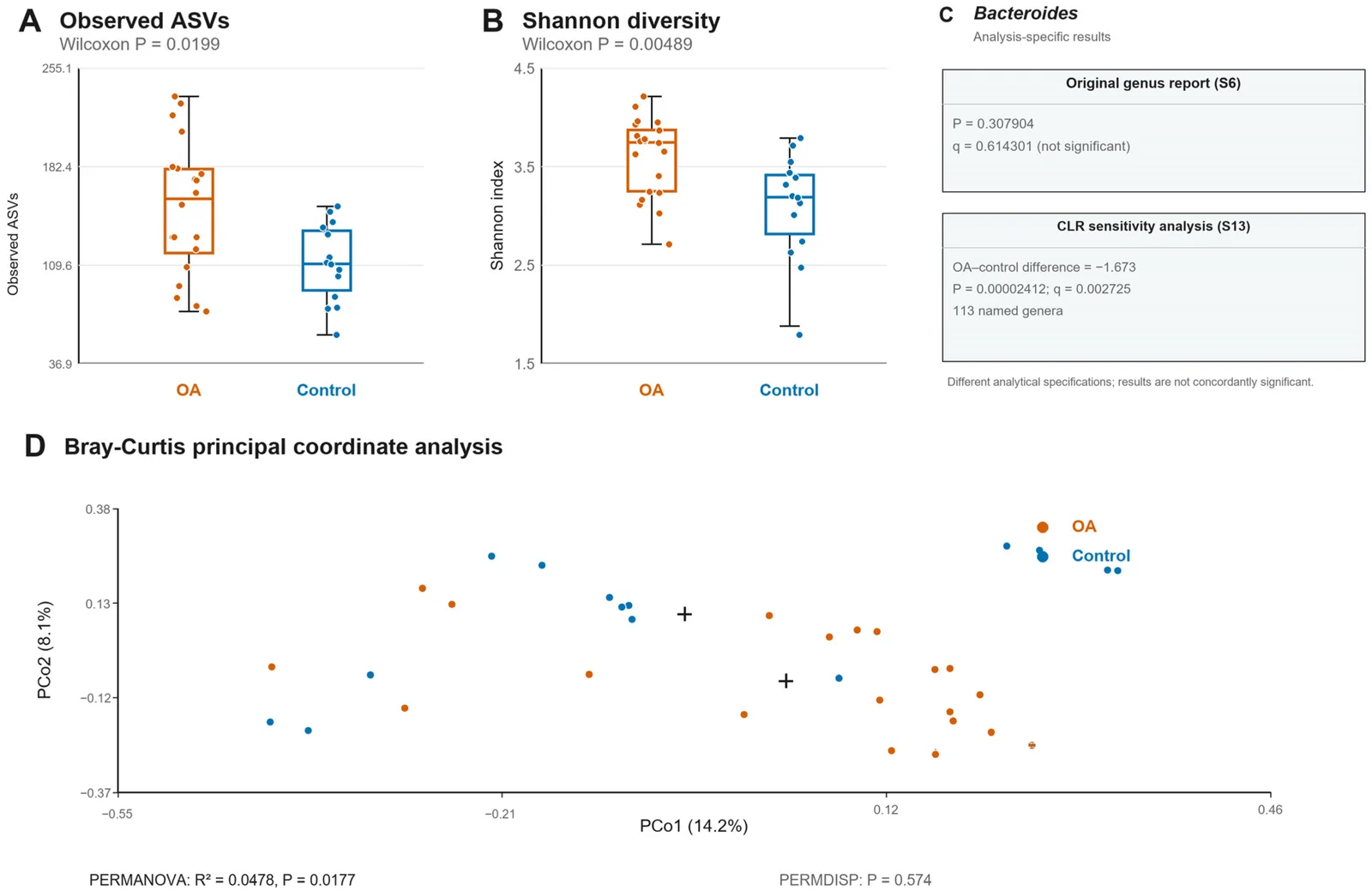
**Gut-microbiota findings in the clinical cohort.** (**A**) Observed amplicon sequence variants. (**B**) Shannon diversity. (**C**) *Bacteroides* results from distinct analytical specifications: the original genus report (Table S6; *p* = 0.307904, q = 0.614301) and the centered log-ratio (CLR) sensitivity analysis across 113 named genera (Table S13; OA–control difference = −1.673, *p* = 0.00002412, q = 0.002725). These analyses are not concordantly FDR-significant. (**D**) Bray–Curtis principal coordinate analysis. Panels (**A**,**B**) show participants and interquartile-range boxes; in (**D**), colored points are participants and black crosses are group centroids. Orange indicates OA and blue indicates controls. PERMANOVA R^2^ = 0.0478, *p* = 0.0177; PERMDISP *p* = 0.574 (9999 permutations each).

The median observed ASV count was 158.5 (117.75–181.25) in OA and 110.5 (89.75–135.75) in controls (*p* = 0.0199; rank-biserial r = 0.479). The median Shannon index was 3.767 (3.256–3.905) in OA and 3.207 (2.819–3.442) in controls (*p* = 0.00489; rank-biserial r = 0.564). Bray–Curtis reanalysis indicated that group explained 4.78% of community-structure variation (PERMANOVA pseudo-F = 1.605, R^2^ = 0.0478, *p* = 0.0177; 9999 permutations), while within-group dispersions were similar (PERMDISP pseudo-F = 0.408, *p* = 0.574; 9999 permutations) (Table 5; Figure 4).

The original genus-level report did not identify an FDR-significant *Bacteroides* difference (*p* = 0.3079, q = 0.6143; Table S6). In contrast, the separate compositional sensitivity analysis across 113 named genera showed a lower *Bacteroides* centered log-ratio value in OA (difference = −1.673; Welch *p* = 0.0000241; q = 0.00273; Table S13). These analyses use different transformations and testing specifications and should not be presented as concordant FDR-significant results. The compositional result supports an exploratory taxonomic association, not a confirmed proteolytic function.

### 3.5. Clinical-Cohort Results: Group-Level Covariation of Bacteroides and Six Nitrogenous Tripeptides

In the directed paired analysis of all 34 participants, the unadjusted Spearman correlation between *Bacteroides* and each FDR-significant tripeptide followed the corresponding between-group direction. Cys-Glu-Phe, Phe-Asn-Asn, and Lys-Ile-Glu, which were higher in OA, correlated inversely with *Bacteroides*; Ile-Leu-Asn, Phe-Phe-Gln, and Gln-Phe-Tyr, which were lower in OA, correlated positively. Absolute correlation coefficients ranged from 0.485 to 0.584, with q values from 0.00171 to 0.00362 (Table 5; Table S7). Thus, lower *Bacteroides* abundance and the putatively annotated tripeptide profile co-occurred within the OA-associated intestinal state.

After OA/control adjustment, the six partial correlation coefficients decreased to absolute values of 0.133–0.302, with q values of 0.457–0.461 (Table S7). None remained significant. Thus, the pooled correlations may largely reflect shared case–control separation. These data do not demonstrate independent *Bacteroides*–peptide relationships or genus-specific proteolysis, substrate uptake, or cross-feeding; the small cohort also limits precision for residual associations.

### 3.6. Cross-Source Integration: Synovial SLC1A5 and Clinical Amino Acid Metabolic Features

**Figure 5:**
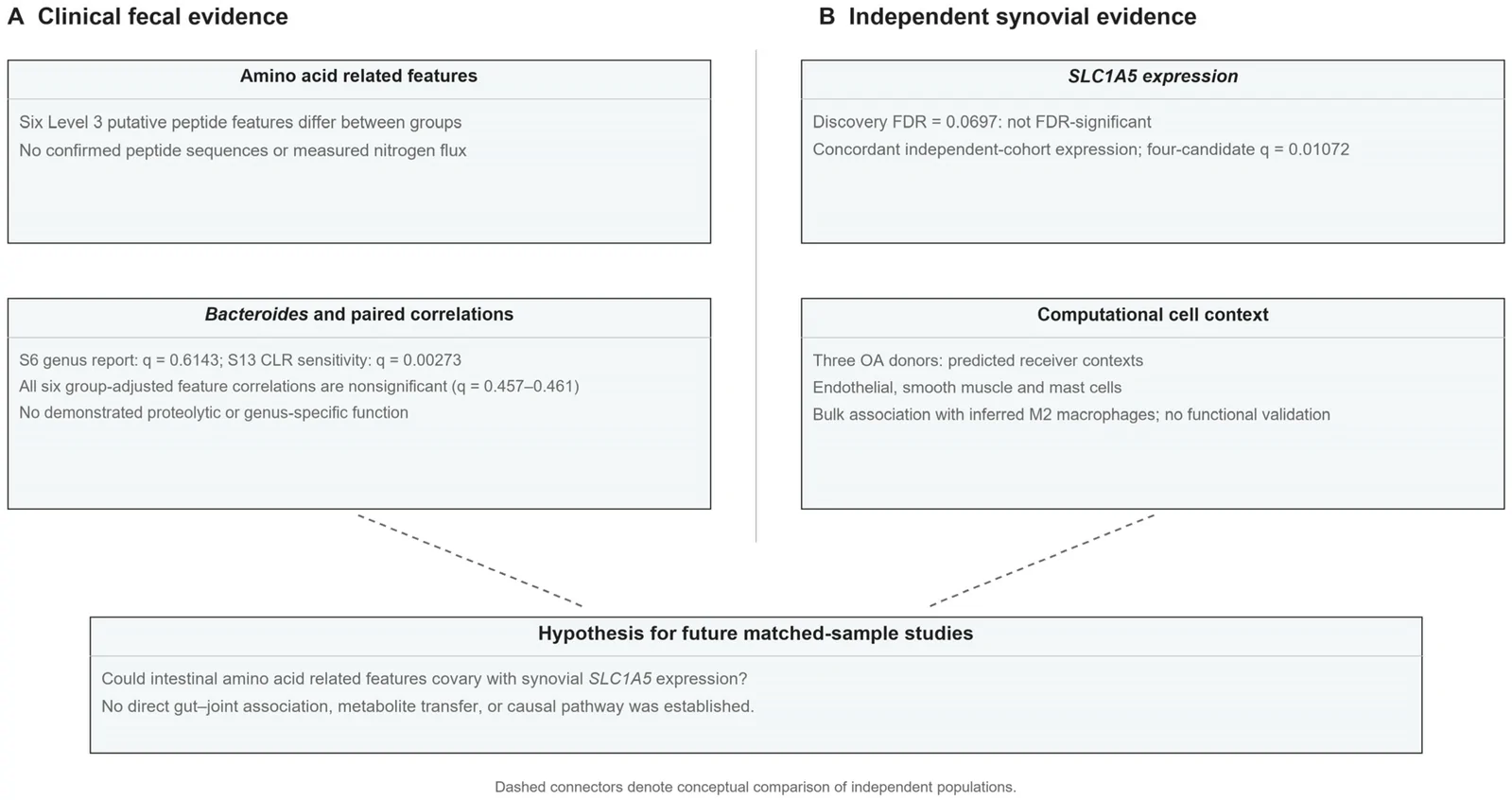
**Hypothesis-generating comparison of independent evidence streams.** (**A**) The clinical cohort showed a lower *Bacteroides*-centered log-ratio value and differences in six features with putative tripeptide annotations; none of their group-adjusted correlations was significant. (**B**) Independent synovial datasets provided *SLC1A5* expression and computational cell-context findings. Any proposed link between intestinal metabolism and synovial transport remains untested. Diagram connections must not be interpreted as measured metabolite transfer, confirmed proteolysis, or causal direction.

Public synovial analyses prioritized *SLC1A5* as an exploratory candidate and described its computational cell context and bulk immune associations. Independently, the clinical cohort showed a lower *Bacteroides*-centered log-ratio value and differences in six features with putative Gln/Asn/Glu tripeptide annotations. The fecal genus–feature correlations were attenuated after group adjustment. Because the synovial and fecal data came from different participants, no direct gut–joint association, mediation effect, or direction of influence was tested. Figure 5 presents only a hypothesis-generating framework for future matched-sample studies.

## 4. Discussion

This study brings together independent observations from public synovial transcriptomes and a small clinical fecal multi-omics cohort. Cross-model screening prioritized four genes, including *SLC1A5*, whose expression was directionally concordant in a second synovial cohort. In the clinical samples, six features carrying Level 3 tripeptide annotations differed between groups, whereas free Gln, Asn, and Glu did not reach FDR significance. *Bacteroides* showed a lower centered log-ratio value in OA, although the original genus report was not FDR-significant, but its correlations with the six features were not significant after group adjustment. These findings motivate a hypothesis; they do not establish a connected intestinal–synovial pathway.

*SLC1A5* was selected through an exploratory multistage procedure. Its discovery FDR was 0.0697, so it should not be described as a discovery-cohort FDR-significant gene. The independent cohort supported the direction of expression (*p* = 0.00536; four-candidate q = 0.01072), but the AUC interval was wide (0.61–0.99). Preselection before cross-validation and reuse of the same discovery samples across algorithms can produce optimistic feature selection; agreement between algorithms does not resolve this risk. Repeated nested cross-validation with selection confined to each training fold, label-permutation testing of the entire pipeline, and additional external cohorts would be needed to evaluate stability and prediction. No such additional model validation is claimed here.

Within the single-cell analysis, endothelial cells had the highest *SLC1A5* sensor_in_receiver intensity, followed by smooth muscle cells and mast cells. The synovial endothelium is the interface through which circulating nutrients enter the tissue and also coordinates leukocyte adhesion, migration, and local vascular remodeling. Human OA synovial single-cell atlases consistently identify an endothelial compartment [24], and histologic studies show that endothelial proliferation, vascular area, and vascular endothelial growth factor (VEGF) expression increase with synovial inflammation and macrophage infiltration [25]. Glutamine metabolism replenishes tricarboxylic acid cycle intermediates in endothelial cells and supports biosynthesis, proliferation, and redox homeostasis [26,27]. These prior findings motivate a possible vascular context for the predicted *SLC1A5* expression pattern, but do not validate transport activity in the present samples. Three OA donors cannot establish donor-level reproducibility, and pooled-cell significance should not be interpreted as independent replication across patients.

*SLC1A5* expression was associated with inferred M2 macrophage proportions after group adjustment. However, bulk deconvolution and single-cell receiver predictions estimate different quantities. They do not demonstrate direct interactions between vascular cells and macrophages or a causal effect on macrophage polarization. Spatial localization and functional perturbation would be required to test these possibilities.

Free amino acids and putatively annotated peptide features provide complementary analytical readouts. Fecal amino acid abundance integrates dietary input, microbial and host metabolism, absorption, and excretion. Although peptide-related signals could be influenced by protein breakdown and peptide handling, a cross-sectional peak-area measurement cannot resolve these processes. In particular, Level 3 annotations do not establish the residue sequence, rule out isomers, or justify interpreting the observed pattern as sequence-selective proteolysis. The feature names are candidate annotations rather than confirmed molecular identities.

The Gln/Asn/Glu-focused analysis was motivated by *SLC1A5* biology and the metabolic connections between glutamine and glutamate. This biological rationale does not imply that the annotated fecal tripeptides are ASCT2 substrates or that glutamate-containing signals demonstrate direct glutamate transport by ASCT2. The main finding is a difference in six putatively annotated features alongside nonsignificant free-amino-acid comparisons. Earlier fecal metabolomics work in obesity-related OA [6] provides context, but targeted structural validation and flux measurements are needed before attributing these signals to nitrogen turnover or synovial amino acid supply.

Some *Bacteroides* species have proteases, peptidases, and peptide-related metabolic capabilities [10,11,12]. This prior evidence cannot be extrapolated to the function of the genus in the present cohort. The original genus report was not FDR-significant for *Bacteroides* (q = 0.6143; Table S6), whereas the separate centered log-ratio analysis across 113 named genera was significant (difference = −1.673; q = 0.00273; Table S13). This dependence on the analytical specification warrants caution. Unadjusted correlations with the six candidate features followed their case–control differences, but group-adjusted q values were 0.457–0.461 (Table S7). We therefore interpret *Bacteroides* as an exploratory taxonomic feature, not a demonstrated marker of proteolytic activity. Metagenomics, metatranscriptomics, and strain-specific experiments would be needed to identify functional contributions.

The contribution of this study is an exploratory comparison of synovial amino acid transport-related expression and paired clinical fecal profiles. Integration across the fecal platforms was performed at the participant level, whereas integration with the synovial datasets was conceptual. The resulting framework generates questions about amino acid metabolism in OA without establishing a common mechanism across tissues.

Several limitations constrain interpretation. The clinical cohort comprised only 20 OA participants and 14 controls. BMI was higher in OA (26.20 versus 25.32 kg/m^2^; *p* = 0.013), and the reported analyses did not comprehensively adjust for BMI, diet, medications, age, sex, or comorbidities. Dietary intake was not quantitatively characterized, and recorded medication categories do not eliminate residual confounding. The high OPLS-DA R^2^Y, incompletely documented resampling, discovery feature preselection, and absence of an independent clinical validation cohort limit predictive claims. The single-cell analysis included only three OA donors, without healthy comparators or donor-level validation. Metabolite identities remained putative and were not confirmed by targeted LC–MS/MS with authentic standards; genus-level 16S data cannot establish species-specific functions. Finally, the synovial and fecal datasets were unmatched, and no circulating metabolite measurements, transport assays, perturbation experiments, or functional gut–joint validation were available. Accordingly, neither a direct gut–joint association nor a causal metabolic pathway can be inferred. Differences between the original genus report and the compositional sensitivity results further limit claims of analytical robustness.

Future work should first confirm candidate peptide identities using authentic standards, retention-time agreement, and diagnostic tandem mass spectra, followed by validated quantitative assays. Larger cohorts with matched fecal, circulating, and synovial measurements and better dietary and medication characterization could test whether these features covary within individuals. Donor-aware single-cell analyses, spatial localization, and functional experiments could then examine the proposed cell contexts and mechanisms. These are validation priorities, not completed experiments.

## 5. Conclusions

*SLC1A5* was prioritized as an exploratory synovial candidate, with a nominal discovery association and a concordant decrease in an independent cohort. A separate clinical cohort showed a lower *Bacteroides*-centered log-ratio value and differences in six putatively annotated peptide features, whose genus associations were not significant after group adjustment. Together, these observations support hypothesis generation about amino acid-related features in OA; they do not establish peptide identities, altered metabolic flux, or a direct gut–synovium pathway.

## Figures and Tables

**Table 1:** Public-data analysis overview.

Analysis Stage	Dataset and Sample	Method and Purpose
Candidate screen	GSE152805 and GSE89408	Intersect metabolite-sensor genes with nominal differential expression.
Model prioritization	GSE89408; 28 healthy and 22 OA	Elastic net, SVM-RFE, and XGBoost; identify a cross-model consensus.
Independent expression assessment	GSE55235; 10 healthy and 10 OA	Check expression direction and describe single-gene ROC AUC.
Cellular context	GSE152805; three OA synovial donors	Seurat annotation and MEBOCOST expression-based communication predictions.
Immune association	GSE89408 bulk synovium	CIBERSORT deconvolution and group-adjusted rank correlations.

Abbreviations: OA, osteoarthritis; ROC, receiver operating characteristic; AUC, area under the ROC curve; SVM-RFE, support vector machine recursive feature elimination; XGBoost, extreme gradient boosting; MEBOCOST, metabolic cell–cell communication modeling using single-cell transcriptomes; CIBERSORT, cell-type identification by estimating relative subsets of RNA transcripts. The datasets are independent of the clinical fecal cohort. Cross-model agreement and predicted cell context are exploratory.

**Table 2:** Demographic and clinical characteristics of the study participants.

Variable	OA Group (n = 20)	Control Group (n = 14)	Between-Group Comparison
Age, years	63.30 ± 5.11	61.71 ± 4.55	*p* = 0.359
Female sex, n (%)	12 (60.0%)	8 (57.1%)	*p* = 1.000
BMI, kg/m^2^	26.20 ± 1.13	25.32 ± 0.62	*p* = 0.013
Kellgren–Lawrence grade, n (%)	Grade 2: 7 (35.0%) Grade 3: 8 (40.0%) Grade 4: 5 (25.0%)	—	Descriptive
Metabolic comorbidities, n (%)	10 (50.0%)	4 (28.6%)	*p* = 0.296
Recent antibiotics/probiotics, n (%)	0 (0%)	0 (0%)	Not applicable

Data are presented as mean ± SD for continuous variables and n (%) for categorical variables. Between-group comparisons were performed using independent-samples *t* tests for continuous variables and Fisher’s exact test for categorical variables. Kellgren–Lawrence grade was summarized descriptively. BMI, body mass index; OA, osteoarthritis.

**Table 3:** Public data screening and independent expression assessment of candidate genes and *SLC1A5*-related context.

Analysis Stage	Dataset/Sample	Method	Main Finding	Role in Study
Metabolite-sensing candidate screen	GSE152805 and GSE89408	Intersection of 25 sensors with nominal DEGs	Eight candidate genes	Reduced candidate space
Machine-learning prioritization	GSE89408: 28 healthy; 22 OA	Elastic net, SVM-RFE, XGBoost	*SLC2A3*, *SLC16A1*, *SLC1A5*, and *ANXA2* shared by all models	Cross-model consensus set
Independent expression assessment	GSE55235: 10 healthy; 10 OA	Differential expression and ROC	*SLC1A5* logFC = −0.695, *p* = 0.00536; four-candidate q = 0.01072, AUC = 0.830; *ANXA2* concordant	Independent cohort support
*SLC1A5* discovery-cohort association	GSE89408	Differential expression and ROC	logFC = −0.532, *p* = 0.0159, FDR = 0.0697, AUC = 0.680	Discovery-cohort direction
Predicted single-cell context	GSE152805: 3 OA samples; 10,547 cells	Cell annotation and MEBOCOST	Eight cell states; 38 *SLC1A5*-related events	Described computational context
Predicted *SLC1A5* receiver context	Endothelial cells; SMCs; mast cells	sensor_in_receiver intensity	1.0445, 0.9708, and 0.8917; 11, 9, and 9 events	Vascular and immune receiving context
Immune-microenvironment association	GSE89408 bulk tissue	Spearman and group-adjusted partial correlation	*SLC1A5*-M2: ρ = −0.624; adjusted r = −0.575; FDR = 0.002	Connected *SLC1A5* to macrophage context

Abbreviations: AUC, area under the receiver operating characteristic (ROC) curve; DEG, differentially expressed gene; FDR, false discovery rate; OA, osteoarthritis; SMC, smooth muscle cell; SVM-RFE, support vector machine recursive feature elimination; XGBoost, extreme gradient boosting. The discovery *SLC1A5* result did not meet FDR < 0.05. In GSE55235, Benjamini–Hochberg correction across the four consensus-gene raw *p* values gave q = 0.00001265 (*SLC2A3*), 0.445824 (*SLC16A1*), 0.010716 (*SLC1A5*), and 0.062243 (*ANXA2*). These are four-candidate q values, not transcriptome-wide FDR. The AUC 95% confidence interval for *SLC1A5* was 0.61–0.99. MEBOCOST events and receiver expression are computational predictions, not measurements of transport activity. Sources: Tables S8–S11.

**Table 4:** Six FDR-significant features with Level 3 putative tripeptide annotations.

Putative Tripeptide Feature	Residue	Main log2FC	Main q	Sensitivity log2FC	Sensitivity q	Annotation
Cys-Glu-Phe	Glu	1.515	0.002454	3.046	0.00644	Level 3
Phe-Asn-Asn	Asn	1.297	0.004030	2.359	0.00697	Level 3
Lys-Ile-Glu	Glu	0.613	0.004306	0.741	0.02143	Level 3
Ile-Leu-Asn	Asn	−5.174	0.005801	−3.924	0.00367	Level 3
Phe-Phe-Gln	Gln	−4.237	0.008148	−3.220	0.00373	Level 3
Gln-Phe-Tyr	Gln	−7.094	0.009476	−4.875	0.00711	Level 3

FDR, false discovery rate; FC, fold change; Gln, glutamine; Asn, asparagine; Glu, glutamate; Cys, cysteine; Phe, phenylalanine; Lys, lysine; Ile, isoleucine; Leu, leucine; Tyr, tyrosine. Main-analysis q values are corrected across 1768 metabolites (Table S2); sensitivity q values across 5967 features (Tables S4 and S12). Candidate names are Level 3 putative annotations and do not confirm exact molecular identity or peptide sequence.

**Table 5:** Main gut microbiota findings in the local clinical cohort.

Analysis	OA Group	Control Group	Effect/Test	Evidence Level
Observed ASVs	158.5 (117.75–181.25)	110.5 (89.75–135.75)	*p* = 0.0199; r = 0.479	Ecological context
Shannon index	3.767 (3.256–3.905)	3.207 (2.819–3.442)	*p* = 0.00489; r = 0.564	Ecological context
Bray–Curtis	—	—	PERMANOVA R^2^ = 0.0478, *p* = 0.0177; PERMDISP *p* = 0.574	Community structure
*Bacteroides*	—	—	Original report q = 0.6143; CLR difference = −1.673, q = 0.00273	Analysis-dependent taxonomic association
Unadjusted *Bacteroides*–tripeptide correlation	Higher-in-OA features: inverse association	Lower-in-OA features: positive association	|r| = 0.485–0.584; q = 0.00171–0.00362	Group-level covariation
Group-adjusted *Bacteroides*–tripeptide correlation	—	—	|partial r| = 0.133–0.302; q = 0.457–0.461	No significant residual association

ASV, amplicon sequence variant; CLR, centered log-ratio; OA, osteoarthritis; PERMANOVA, permutational multivariate analysis of variance; PERMDISP, permutational analysis of multivariate dispersions. Diversity summaries are medians (interquartile ranges); r is the rank-biserial effect size. The original genus report (Table S6) and CLR sensitivity analysis (Table S13) use different analytical specifications and do not give concordant FDR-significant *Bacteroides* results. Unverified mean relative abundances and the original raw-analysis q = 0.00905 are omitted. Exact genus–feature correlation results are in Table S7. Genus-level abundance does not establish proteolytic function.

## Data Availability

The raw data supporting the conclusions of this article will be made available by the authors on request.

## Supplementary Materials

The following supporting information can be downloaded at: https://www.mdpi.com/article/10.3390/metabo16100764/s1, Table S1, clinical sample information; Table S2, main analysis of all 1768 quality-controlled metabolites; Table S3, 347 FDR-significant metabolites; Table S4, log2-Welch results for 24 Level 3 putatively annotated Gln/Asn/Glu-related tripeptide features; Table S5, Level 4 tripeptide candidates; Table S6, genus-level differential analysis; Table S7, unadjusted and group-adjusted correlations between *Bacteroides* and the six tripeptides; Table S8, eight candidate genes and three-model selection results; Table S9, external expression results in GSE55235 and bootstrap AUC intervals (the four-candidate adjustment reported in the revised text is calculated from its raw *p* values); Table S10, 38 predicted *SLC1A5*-related MEBOCOST events; Table S11, raw and group-adjusted correlations between *SLC1A5* and inferred immune-cell proportions; Table S12, full 5967-feature log2-Welch sensitivity analysis; and Table S13, CLR sensitivity analysis across 113 named genera.

## Abbreviations

ACR	American College of Rheumatology
ASCT2	alanine, serine, cysteine transporter 2
ASV	amplicon sequence variant
AUC	area under the receiver operating characteristic curve
BMI	body mass index
bp	base pair
CI	confidence interval
CIBERSORT	cell-type identification by estimating relative subsets of RNA transcripts
CLR	centered log-ratio
CTAB	cetyltrimethylammonium bromide
cum	cumulative
DEG	differentially expressed gene
DNA	deoxyribonucleic acid
FC	fold change
FDR	false discovery rate
FLASH	Fast Length Adjustment of SHort reads
GEO	Gene Expression Omnibus
IQR	interquartile range
K-L	Kellgren–Lawrence
KNN	k-nearest neighbors
LC–MS/MS	liquid chromatography–tandem mass spectrometry
logFC and log2FC	base-2 logarithm of fold change
MEBOCOST	metabolic cell–cell communication modeling using single-cell transcriptomes
MS1	full-scan mass spectrometry
MS2 and MS/MS	tandem mass spectrometry
MSI	Metabolomics Standards Initiative
mTOR	mechanistic target of rapamycin
mTORC1	mTOR complex 1
*m*/*z*	mass-to-charge ratio
OA	osteoarthritis
OPLS-DA	orthogonal partial least squares discriminant analysis
PCA	principal component analysis
PCoA	principal coordinate analysis
PERMANOVA	permutational multivariate analysis of variance
PERMDISP	permutational analysis of multivariate dispersions
ProlC	proliferative-state cells
QC	quality control
QIIME 2	Quantitative Insights Into Microbial Ecology 2
Q^2^	cross-validated predictive ability
RNA	ribonucleic acid
ROC	receiver operating characteristic
rRNA	ribosomal RNA
R^2^X and R^2^Y	explained variance in predictors and response, respectively
SD	standard deviation
SDS	sodium dodecyl sulfate
SIF	synovial intimal fibroblast
*SLC1A5*	solute carrier family 1 member 5
SMC	smooth muscle cell
SSF	synovial subintimal fibroblast
STROBE	Strengthening the Reporting of Observational Studies in Epidemiology
STORMS	Strengthening The Organization and Reporting of Microbiome Studies
SVM-RFE	support vector machine recursive feature elimination
TMM	trimmed mean of M-values
UMAP	uniform manifold approximation and projection
VEGF	vascular endothelial growth factor
XGBoost	extreme gradient boosting
Amino acid codes	
Ala	alanine
Arg	arginine
Asn	asparagine
Asp	aspartate
Cys	cysteine
Gln	glutamine
Glu	glutamate
Gly	glycine
His	histidine
Ile	isoleucine
Leu	leucine
Lys	lysine
Met	methionine
Phe	phenylalanine
Pro	proline
Ser	serine
Thr	threonine
Trp	tryptophan
Tyr	tyrosine
Val	valine
*ANXA2*	annexin A2
*LRRC8C*	leucine-rich repeat containing 8 VRAC subunit C
*PMVK*	phosphomevalonate kinase
*RARG*	retinoic acid receptor gamma
*SLC16A1*	solute carrier family 16 member 1
*SLC2A3*	solute carrier family 2 member 3
*SLC44A1*	solute carrier family 44 member 1
